# Mortality Among Blacks or African Americans with HIV Infection — United States, 2008–2012

**Published:** 2015-02-06

**Authors:** Azfar-e-Alam Siddiqi, Xiaohong Hu, H. Irene Hall

**Affiliations:** 1 Division of HIVAIDS Prevention, National Center for HIV/AIDS, Viral Hepatitis, STD, and TB Prevention, CDC

A primary goal of the National HIV/AIDS Strategy is to reduce HIV-related health disparities, including HIV-related mortality in communities at high risk for human immunodeficiency virus (HIV) infection (1). As a group, persons who self-identify as blacks or African Americans (referred to as blacks in this report), have been affected by HIV more than any other racial/ethnic population. Forty-seven percent of persons who received an HIV diagnosis in the United States in 2012 and 43% of all persons living with diagnosed HIV infection in 2011 were black. Blacks also experienced a low 3-year survival rate among persons with HIV infection diagnosed during 2003–2008 (2). CDC and its partners have been pursuing a high-impact prevention approach and supporting projects focusing on minorities to improve diagnosis, linkage to care, and retention in care, and to reduce disparities in HIV-related health outcomes (3). To measure trends in disparities in mortality among blacks, CDC analyzed data from the National HIV Surveillance System. The results of that analysis indicated that among blacks aged ≥13 years the death rate per 1,000 persons living with diagnosed HIV decreased from 28.4 in 2008 to 20.5 in 2012. Despite this improvement, in 2012 the death rate per 1,000 persons living with HIV among blacks was 13% higher than the rate for whites and 47% higher than the rate for Hispanics or Latinos. These data demonstrate the need for implementation of interventions and public health strategies to further reduce disparities in deaths.

Data from the National HIV Surveillance System for 2008–2012 and reported to CDC through June 2014 were used to determine the numbers of deaths and rates of death among black persons living with HIV aged ≥13 years at the time of death. Numbers and rates for the total U.S. population and for whites and Hispanics or Latinos were calculated for comparison. Two sets of death rates were calculated overall and by age, race/ethnicity and sex: 1) deaths per 100,000 population and 2) deaths per 1,000 persons living with HIV. The numerator for each rate was the estimated number of deaths by year of death. The denominators for the rates per 100,000 population were calculated using year-specific census or postcensus data (for persons aged ≥13 years) from the U.S. Census Bureau for the years 2008–2012 (4). For a given year (year X), the denominator for the rate per 1,000 persons living with HIV was calculated by adding the number of new HIV diagnoses among persons aged ≥13 years during year X to the number of persons living with diagnosed HIV aged ≥13 years at the end of the year X-1. For rates by HIV transmission category, only rates per 1,000 persons living with HIV could be calculated because the U.S. Census does not collect the data needed for calculating rates per 100,000 population. The number of deaths was statistically adjusted for reporting delays and missing transmission category (5).

In 2012, an estimated 8,165 (48%) deaths occurred among black persons living with HIV, which was 1.5 times the number of deaths among whites (5,426) and 3.2 times the deaths among Hispanics or Latinos (2,586). During 2008–2012, there was a consistent decline in the number of deaths and rates of death among blacks. The number of deaths decreased 18%, and rate per 100,000 population decreased 21%; rate per 1,000 persons living with HIV decreased 28%. Although deaths also decreased among other race/ethnicity groups, the decreases generally were greater and more consistent among blacks than among other races/ethnicities (Table 1).

In 2012, deaths per 1,000 persons living with HIV among blacks were higher among older persons compared with younger persons, with the highest rate (41.3) among those aged ≥55 years. By transmission category, among black males, the lowest death rate (per 1,000 persons living with HIV) was among males whose HIV infection was attributed to male-to-male sexual contact (15.3), and the highest rate was among males who had their HIV infection attributed to injection drug use (33.1). Among black females, the death rate among those with HIV infection attributed to heterosexual contact (17.9) also was lower compared with the rate among those black females with infection attributed to injection drug use (29.2). These patterns were consistent across all races/ethnicities (Table 2).

Racial/ethnic disparities varied among states. In 23 states and the District of Columbia, the death rate per 1,000 persons living with HIV in blacks was higher than that in whites, whereas in 27 states blacks had a death rate that was lower than that in whites. The rate among blacks was higher than that among Hispanics/Latinos in 37 states and the District of Columbia. In 2012, the highest and lowest rates per 1,000 persons living with HIV among blacks were in West Virginia (28.9) and Nebraska (9.3), respectively, and among the 10 states with the highest death rates per 1,000 persons living with HIV in blacks, seven were in the South. The highest and lowest rates per 100,000 population among blacks were in the District of Columbia (98.4) and Alaska (5.2), respectively, and the largest number of deaths (1,147) occurred in Florida (Table 3).

## Discussion

The results of these analyses indicate that black persons living with HIV experienced higher numbers and rates of deaths during 2008–2012 than other races/ethnicities. However, the numbers and rates of death declined consistently during the same period. The death rate per 1,000 persons living with HIV among blacks decreased 28% during 2008–2012, more than the overall decline (22%) seen among all persons living with HIV. Other than among blacks, such a consistent decline was observed only among Hispanics or Latinos.

Despite differences in the magnitude of the death rates, the mortality pattern among blacks by age, sex, and transmission category was similar to that seen in other races/ethnicities. In all three races/ethnicities, the highest rates of death were observed in the oldest persons living with HIV infection (aged ≥55 years), who might have been living longer with HIV and had more complications from HIV, and who also might have a higher all-cause mortality because of their age. By transmission category, in all races/ethnicities, men who have sex with men had lower death rates than persons in most other transmission categories; whereas persons who had their infection attributed to injection drug use had the highest death rate. This finding is consistent with reports that persons who use injection drugs are more likely to have comorbid conditions and an increased all-cause mortality than nonusers of injection drugs (6).

Whereas the overall disparity in deaths per 1,000 blacks living with HIV compared with whites living with HIV has narrowed over the period covered by this analysis (from 37% in 2008 to 13% in 2012), in 2012, the death rate was still higher (20.5) among blacks compared with whites (18.1) and Hispanics or Latinos (13.9). In general, blacks with HIV are less likely to have their infection diagnosed, with 15% unaware of their infection in 2011 compared with 12% of whites (7). Among blacks whose HIV was diagnosed in 2012, 77% were linked to care, which was lower than the percentage among any other race/ethnicity; in 2011, the percentages of black persons living with HIV who were retained in care (48%) or who had a suppressed viral load (40%) were lower than the percentages among whites and Hispanics or Latinos (7).

The findings in this report are subject to at least one limitation. The report evaluates all-cause mortality in persons living with HIV and does measure mortality resulting from HIV. Therefore, the report does not allow for any direct evaluation of possible differences in quality of care among persons living with HIV, by race/ethnicity. However, because HIV infection causes immune suppression, which in turn results in fatal comorbidities such as cancers and opportunistic infections, all-cause mortality likely is a better indicator of the actual mortality experience than cause-specific mortality.

What is already known on this topic?

In 2012, blacks accounted for 47% of persons who received a human immunodeficiency virus (HIV) diagnosis, and in 2011, they accounted for 43% of persons living with HIV. During 2008–2011 more deaths among black persons living with HIV occurred each year than among any other race/ethnicity.

What is added by this report?

During 2009–2012, the number of deaths among black persons living with HIV declined 18%, and the rate of death per 1,000 persons living with HIV declined 28%. In 2012, the number of deaths per 1,000 black persons living with HIV was 20.5 among blacks compared with 18.1 among whites and 13.9 among Hispanics or Latinos.

What are the implications for public health practice?

To achieve the National HIV/AIDS strategy’s objective of reducing health disparities, efforts are needed to increase entry into and retention in care of black persons living with diagnosed HIV. Rates of death caused by HIV infection vary by geographic area, and efforts tailored to each area’s unique needs and situations might be needed to reduce the rates of early deaths among blacks.

CDC, with its partners, has been pursuing a high-impact prevention approach to advance the goals of the National HIV/AIDS Strategy and to maximize the effectiveness of current HIV prevention and care methods (3). CDC also supports projects focused on blacks aimed at optimizing outcomes along the continuum of care, such as HIV testing (the first essential step for entry into the continuum of care) and projects that support linkage to, retention in, and return to care for all persons infected with HIV (8). The results of the analyses in this report show that, although disparities in mortality by race/ethnicity persist, the overall outlook for all persons living with HIV has improved, and the gaps between different races/ethnicities have narrowed. Focusing prevention and care efforts on minority populations with a disproportionate HIV burden could lead to further reduction, if not elimination, of health disparities, such as higher mortality, and help achieve the goals of the National HIV/AIDS Strategy.

## Figures and Tables

**TABLE 1:** Estimated number and rate of deaths of persons aged ≥13 years with diagnosed HIV infection,* by race/ethnicity — United States, 2008–2012

Race/Ethnicity	2008			2009			2010			2011			2012		
				
	No.†	Rate per 100,000 pop.	Rate per 1,000 PLWH§	No.†	Rate per 100,000 pop.	Rate per 1,000 PLWH§	No.†	Rate per 100,000 pop.	Rate per 1,000 PLWH§	No.†	Rate per 100,000 pop.	Rate per 1,000 PLWH§	No.†	Rate per 100,000 pop.	Rate per 1,000 PLWH§
Black/African American	9,920	33.1	28.4	9,596	31.7	26.5	8,682	28.3	23.3	8,444	27.2	21.9	8,165	26.0	20.5
Hispanic/Latino	2,949	8.5	18.5	2,913	8.2	17.5	2,809	7.4	16.3	2,799	7.2	15.6	2,586	6.5	13.9
White	5,662	3.3	20.8	5,545	3.2	19.8	5,395	3.2	18.8	5,307	3.1	18.1	5,426	3.2	18.1
Other races	890	5.5	22.8	998	6.1	24.6	1,003	5.5	23.9	946	5.0	21.8	989	5.1	22.0
**Total**	**19,421**	**7.7**	**23.7**	**19,052**	**7.5**	**22.5**	**17,890**	**7.0**	**20.5**	**17,496**	**6.8**	**19.4**	**17,166**	**6.6**	**18.5**

**Abbreviations:** HIV = human immunodeficiency virus; PLWH = persons living with diagnosed HIV infection.

* Data include persons with diagnosed HIV infection regardless of stage of disease at diagnosis. Deaths of persons with a diagnosis of HIV infection might have resulted from any cause.

† Estimates include statistical adjustment that accounted for reporting delays, but not for incomplete reporting.

§ Rate per 1,000 population aged ≥13 years living with diagnosed HIV infection (PLWH). Denominator was estimated as (no. PLWH at the end of [year X-1]) + (no. new diagnoses during year X).

**TABLE 2:** Estimated number and rate of deaths of persons aged ≥13 years with diagnosed HIV infection,* by race/ethnicity and selected characteristics — United States, 2012

Characteristic	Black/African American			Hispanic/Latino			White			Total†		
			
	No.§	Rate per 100,000 pop.	Rate per 1,000 PLWH¶	No.§	Rate per 100,000 pop.	Rate per 1,000 PLWH¶	No.§	Rate per 100,000 pop.	Rate per 1,000 PLWH¶	No.§	Rate per 100,000 pop.	Rate per 1,000 PLWH¶
**Age at death (yrs)**												
13–24	141	1.8	4.7	33	0.3	3.7	30	0.1	4.1	215	0.4	4.4
25–34	710	13.0	10.8	205	2.4	6.4	254	1.0	8.2	1,227	2.9	9.0
35–44	1,324	26.0	14.1	417	5.5	7.9	788	3.2	11.7	2,696	6.7	11.9
45–54	2,698	50.0	21.0	927	15.7	15.4	2,160	7.2	17.9	6,123	13.8	18.9
≥55	3,292	41.8	41.3	1,003	14.8	31.5	2,194	3.5	29.6	6,905	8.4	35.7
**Transmission category**												
Males												
Male-to-male sexual contact	2,257	—	15.3	940	—	9.4	3,189	—	15.1	6,777	—	14.0
Injection drug use	1,448	—	33.1	584	—	26.1	530	—	33.8	2,723	—	32.1
Male-to-male sexual contact and injection drug use	456	—	27.7	215	—	22.0	530	—	24.3	1,303	—	25.3
Heterosexual contact	1,182	—	24.6	205	—	14.8	254	—	26.7	1,719	—	23.2
Other**	40	—	12.2	18	—	12.4	57	—	25.5	124	—	17.0
**Subtotal**	**5,383**	**36.4**	**20.8**	**1,962**	**9.7**	**13.3**	**4,559**	**5.5**	**17.5**	**12,645**	**9.9**	**18.0**
**Females**												
Injection drug use	847	—	29.2	251	—	26.3	401	—	30.8	1,611	—	29.7
Heterosexual contact	1,894	—	17.9	359	—	12.9	451	—	17.5	2,832	—	17.0
Other**	42	—	11.6	15	—	11.7	14	—	13.3	79	—	12.6
**Subtotal**	**2,782**	**16.7**	**20.1**	**625**	**3.2**	**16.2**	**866**	**1.0**	**21.7**	**4,521**	**3.4**	**19.9**
**Total**	**8,165**	**26.0**	**20.5**	**2,586**	**6.5**	**13.9**	**5,426**	**3.2**	**18.1**	**17,166**	**6.6**	**18.5**

**Abbreviations:** HIV = human immunodeficiency virus; PLWH = persons living with diagnosed HIV infection.

* Data include persons with diagnosed HIV infection regardless of stage of disease at diagnosis. Deaths of persons with a diagnosis of HIV infection might have resulted from any cause.

† Includes other races.

§ Estimates include statistical adjustment that accounted for reporting delays and missing transmission category, but not for incomplete reporting.

¶ Rate per 1,000 population aged ≥13 years living with diagnosed HIV infection (PLWH). Denominator was estimated as (no. PLWH at the end of [year X-1]) + (no. new diagnoses during year X).

** Includes hemophilia, blood transfusion, perinatal exposure, and risks factor not reported or not identified.

**TABLE 3:** Estimated number and rate of deaths of persons aged ≥13 years with diagnosed HIV infection,* by race/ethnicity and state/area of residence — United States, 2012

State/Area	Black/African American			Hispanic/Latino			White			Total†		
			
	No.§	Rate per 100,000 pop.	Rate per 1,000 PLWH¶	No.§	Rate per 100,000 pop.	Rate per 1,000 PLWH¶	No.§	Rate per 100,000 pop.	Rate per 1,000 PLWH¶	No.§	Rate per 100,000 pop.	Rate per 1,000 PLWH¶
Alabama	212	20.7	26.8	4	2.6	10.7	103	3.8	28.9	333	8.3	27.1
Alaska	1	5.2	14.5	1	4.1	21.1	7	1.8	23.2	13	2.3	21.1
Arizona	26	12.4	19.7	45	3.1	12.0	134	4.1	19.0	223	4.1	17.2
Arkansas	40	11.3	18.0	3	2.3	11.3	49	2.6	20.3	99	4.1	19.4
California	369	20.0	17.6	468	4.2	11.7	831	6.3	16.2	1,786	5.7	14.9
Colorado	15	9.5	9.5	28	3.4	12.0	56	1.8	7.6	106	2.5	9.1
Connecticut	72	25.1	21.1	46	11.8	13.7	77	3.5	22.5	200	6.6	19.1
Delaware	47	29.8	24.4	3	4.5	10.8	10	1.9	11.7	60	7.8	19.6
District of Columbia	255	98.4	21.7	17	34.1	17.0	19	9.4	7.8	298	54.0	18.9
Florida	1,147	47.6	23.5	306	8.4	14.7	538	5.5	18.8	2,047	12.4	20.3
Georgia	442	18.3	16.5	23	3.5	6.5	128	2.7	16.3	613	7.6	15.4
Hawaii	1	6.7	13.0	2	2.4	10.1	9	3.2	7.5	20	1.7	8.3
Idaho	0	0.0	0.0	0	0.0	0.0	20	1.8	29.3	20	1.5	22.9
Illinois	270	18.1	16.1	62	4.0	10.2	154	2.2	15.0	530	5.0	15.1
Indiana	65	13.7	19.2	6	2.2	8.8	106	2.4	21.3	187	3.5	19.8
Iowa	6	8.2	16.6	0	0.0	0.0	25	1.1	19.8	33	1.3	17.5
Kansas	14	10.4	18.8	4	2.0	10.4	26	1.4	17.3	49	2.1	17.2
Kentucky	50	18.0	26.4	2	2.5	7.4	79	2.5	23.9	135	3.7	23.5
Louisiana	337	28.8	26.1	7	4.0	8.3	114	4.9	23.7	471	12.4	24.9
Maine	0	0.0	0.0	0	0.0	0.0	8	0.8	8.5	10	0.9	8.2
Maryland	555	39.2	23.2	27	7.1	16.7	103	3.8	21.7	736	15.0	23.2
Massachusetts	54	15.2	9.7	63	12.2	13.4	121	2.8	14.8	244	4.3	12.8
Michigan	223	19.8	26.0	9	2.8	12.7	96	1.5	19.4	344	4.2	22.9
Minnesota	29	13.8	12.3	5	2.7	7.5	57	1.5	15.5	96	2.2	13.5
Mississippi	175	20.0	26.6	7	11.6	30.7	48	3.3	26.8	235	9.6	26.1
Missouri	94	16.9	18.2	6	3.4	9.0	115	2.8	19.8	222	4.4	18.6
Montana	0	0.0	0.0	1	4.9	62.0	4	0.6	13.5	6	0.7	14.0
Nebraska	4	6.9	9.3	1	0.9	4.7	13	1.0	12.8	22	1.4	12.0
Nevada	42	23.2	25.3	35	6.4	20.9	75	5.9	20.2	159	7.0	21.2
New Hampshire	0	0.0	0.0	3	8.6	17.4	12	1.2	15.0	16	1.4	13.6
New Jersey	416	44.1	22.4	141	10.9	14.4	155	3.5	19.3	759	10.2	19.5
New Mexico	3	10.9	25.7	24	3.2	19.0	16	2.2	17.3	54	3.2	20.7
New York	1,081	45.5	20.2	731	25.9	16.9	372	3.8	14.0	2,409	14.6	18.3
North Carolina	351	20.6	20.1	15	2.5	8.4	148	2.7	21.8	541	6.7	20.1
North Dakota	0	0.0	0.0	0	0.0	0.0	2	0.4	15.8	2	0.4	10.6
Ohio	137	12.1	16.4	12	4.4	11.9	189	2.4	21.0	347	3.6	18.3
Oklahoma	26	11.4	20.2	5	2.1	10.6	69	3.1	22.6	117	3.7	21.5
Oregon	6	10.1	15.5	9	2.7	12.9	78	3.0	19.4	98	3.0	18.4
Pennsylvania	321	29.2	20.5	89	15.4	18.0	243	2.8	24.5	701	6.5	21.9
Rhode Island	7	14.1	13.2	2	1.9	3.8	8	1.1	8.1	18	1.9	8.3
South Carolina	216	20.4	19.8	6	3.5	10.6	67	2.6	18.8	291	7.4	18.9
South Dakota	0	0.0	0.0	0	0.0	0.0	5	0.8	18.0	7	1.0	15.3
Tennessee	247	28.4	26.6	4	1.9	6.3	178	4.3	30.2	437	8.1	26.8
Texas	514	21.0	19.2	323	4.4	14.7	456	4.6	21.0	1,394	6.6	19.0
Utah	2	10.1	10.1	1	0.5	2.5	16	0.9	9.2	24	1.1	9.4
Vermont	0	0.0	0.0	0	0.0	0.0	3	0.5	6.6	4	0.7	7.9
Virginia	234	18.2	17.8	26	5.1	15.7	99	2.2	14.9	377	5.5	16.9
Washington	17	8.4	10.4	6	1.0	4.1	134	3.1	18.0	172	3.0	15.2
West Virginia	13	23.7	28.9	2	12.1	37.1	16	1.1	15.0	32	2.0	19.6
Wisconsin	27	9.9	13.3	6	2.6	9.6	29	0.7	11.5	65	1.4	11.8
Wyoming	0	0.0	0.0	0	0.0	0.0	5	1.1	27.4	5	1.0	19.2
**Total**	**8,165**	**26.0**	**20.5**	**2,586**	**6.5**	**13.9**	**5,426**	**3.2**	**18.1**	**17,166**	**6.6**	**18.5**

**Abbreviations:** HIV = human immunodeficiency virus; PLWH = persons living with diagnosed HIV infection.

* Data include persons with diagnosed HIV infection regardless of stage of disease at diagnosis. Deaths of persons with a diagnosis of HIV infection might have resulted from any cause.

† Includes other races.

§ Estimates include statistical adjustment that accounted for reporting delays, but not for incomplete reporting.

¶ Rate per 1,000 population aged ≥13 years living with diagnosed HIV infection (PLWH). Denominator was estimated as (no. PLWH at the end of [year X-1]) + (no. new diagnoses during year X).
